# A New Assistance Navigation Method for Substation Inspection Robots to Safely Cross Grass Areas

**DOI:** 10.3390/s23229201

**Published:** 2023-11-15

**Authors:** Qiang Yang, Song Ma, Gexiang Zhang, Kaiyi Xian, Lijia Zhang, Zhongyu Dai

**Affiliations:** 1 School of Automation, Chengdu University of Information Technology, Chengdu 610225, China; yangqiang@cuit.edu.cn (Q.Y.); masong0007@163.com (S.M.); zlj907674@163.com (L.Z.); 2 Key Laboratory of Natural Disaster Monitoring & Early Warning and Assessment of Jiangxi Province, Jiangxi Normal University, Nanchang 330022, China; 3 Chengdu HitoAI Automatic Control Technology Co., Ltd., Chengdu 610000, China; xky183@163.com; 4 School of Mechanical Engineering, Xihua University, Chengdu 611756, China; dyz7313@126.com

**Keywords:** inspection robot, grass recognition, assistant navigation algorithm, faster-RCNN

## Abstract

With the development of intelligent substations, inspection robots are widely used to ensure the safe and stable operation of substations. Due to the prevalence of grass around the substation in the external environment, the inspection robot will be affected by grass when performing the inspection task, which can easily lead to the interruption of the inspection task. At present, inspection robots based on LiDAR sensors regard grass as hard obstacles such as stones, resulting in interruption of inspection tasks and decreased inspection efficiency. Moreover, there are inaccurate multiple object-detection boxes in grass recognition. To address these issues, this paper proposes a new assistance navigation method for substation inspection robots to cross grass areas safely. First, an assistant navigation algorithm is designed to enable the substation inspection robot to recognize grass and to cross the grass obstacles on the route of movement to continue the inspection work. Second, a three-layer convolutional structure of the Faster-RCNN network in the assistant navigation algorithm is improved instead of the original full connection structure for optimizing the object-detection boxes. Finally, compared with several Faster-RCNN networks with different convolutional kernel dimensions, the experimental results show that at the convolutional kernel dimension of 1024, the proposed method in this paper improves the mAP by 4.13% and the mAP is 91.25% at IoU threshold 0.5 in the range of IoU thresholds from 0.5 to 0.9 with respect to the basic network. In addition, the assistant navigation algorithm designed in this paper fuses the ultrasonic radar signals with the object recognition results and then performs the safety judgment to make the inspection robot safely cross the grass area, which improves the inspection efficiency.

## 1. Introduction

Substations are the hub of the power transmission process, which is an indispensable part of ensuring the safe transmission of power [1]. To ensure the safe operation of the substation, it is necessary to carry out regular inspections to detect the equipment condition [2,3]. The traditional method of substation inspection is manual inspection, where the manual recording of data is inefficient, and recording errors are unavoidable, thus affecting the safe operation of the substation [4,5]. With the development of the smart grid, the use of inspection robots instead of manual inspection has become the mainstream way, which has been widely used in substations [6,7]. To complete the task of inspecting substations, inspection robots need to have autonomous navigation capabilities [8]. At present, inspection robots equipped with 3D LiDAR cannot recognize the grass category, so they treat the grass as an obstacle based on the information of the ultrasonic sensor [9]. Thus, substation inspection robots need to recognize the grass in the substation and safely cross the grass areas to ensure efficient inspections.

Currently, grass recognition methods are mainly divided into two categories: the traditional image-recognition method [10,11] and the recognition method based on deep learning [12,13,14].

The traditional image-recognition methods involve first preprocessing the collected images to eliminate all the features that are not related to grass, then manually designing the features and transforming them into computable vectors or matrices, and finally building a huge feature database, which is used for grass recognition by matching the newly collected image data with the features in the database. After that, the image preprocessing methods are mainly grayscaling [15], median filtering [16], and threshold segmentation [17] to obtain an image containing only grass. Furthermore, the feature extraction is mainly through SIFT (Scale Invariant Feature Transform) [18], SURF (Speeded-Up Robust Features) [19], ORB (Oriented FAST and Rotated BRIEF) [20], and other algorithms to extract features from the pre-processed image. Ref. [17] used edge detection and recognition of grass presence by thresholding. Ref. [21] provided a multi-type feature fusion technique for grass identification, such as different methods in terms of color, shape, and texture feature extraction. These traditional image-recognition methods can achieve grass recognition with manually designed feature extractors and the help of experienced workers. Hence, the level of intelligence in traditional image processing methods for grass recognition is low, and needs to be further improved.

With the development of artificial intelligence technology, object detection using deep-learning methods is widely used [22,23,24]. Another type of recognition method based on deep learning, compared with traditional image processing methods, does not require manual design of features and does not require additional feature extractors. It only needs to learn features from many data samples to recognize grass automatically [25,26,27]. Ref. [28] proposed a hybrid deep convolutional neural network (CNN-SVM) to improve the recognition accuracy of grass in winter oilseed rape-sown fields. Ref. [29] was based on the DeepLab V3+ deep-learning method for grass recognition using RGB images. Ref. [30] used a Single Shot multibox Detector (SSD) to recognize grass in a rice field, which was effective in improving the rice yield. Ref. [31] was based on the deep-learning YOLOv4 model, which introduced an attention mechanism and an adaptive spatial feature fusion structure for grass detection. These methods have achieved better performance for grass recognition in images compared to traditional image processing methods. However, there is still room for improvement in these methods, in which the detection boxes for grass recognition suffer from inaccuracies when multiple objects are present in the image. In addition, although grass recognition has rarely been applied to substation inspection robots, the integration of information from ultrasonic radar and object recognition for scheduling inspection robots to undertake inspection tasks remains to be resolved.

To address the above-mentioned issues, this paper proposes an inspection robot assistant navigation algorithm. Meanwhile, an improved Faster-RCNN network for grass recognition in substations is proposed in this paper. First, an assistant navigation algorithm is designed to fuse the ultrasonic radar signals with the grass recognition results from the improved network and make safety judgments to assist the inspection robot in safely crossing the grass areas. In addition, the strategy to improve the Faster-RCNN network uses a three-layer convolution operation to replace the full connection operation, and in the convolution structure, the advantages of the ResNet [32] network are integrated to form a residual structure, where the improved Faster-RCNN network is named Faster-R101-Conv1024 network. The Faster-R101-Conv1024 network can recognize the object-detection boxes of grass more accurately in the presence of multiple objects in the grass image. Finally, it has been experimentally verified that the method proposed in this paper has better effectiveness in recognizing grass in substations with the combination of inspection robots to cross grassy areas safely.

The contributions of this paper are summarized as follows:

(1) An assistant navigation algorithm for substation inspection robots is designed to fuse ultrasonic radar signals with object recognition results and perform safety determination, which enables the inspection robots to cross the grass on routes of travel safely. The proposed assistant navigation algorithm is transplanted into the embedded platform, which can be effectively combined with the substation inspection robot.
(2) In the assistant navigation algorithm, an improved Faster-RCNN-based network for recognizing the grass of substations is proposed in this paper, which designs a three-layer convolutional structure to replace the full connection structure to improve the accuracy of multiple object-detection boxes in grass images.

The rest of this paper is organized as follows. Section 2 describes the methodology proposed in this paper. The experimental results and analysis are discussed in Section 3. Finally, the conclusions and future work are presented in Section 4.

## 2. The Proposed Method of This Paper

**Figure 1:**
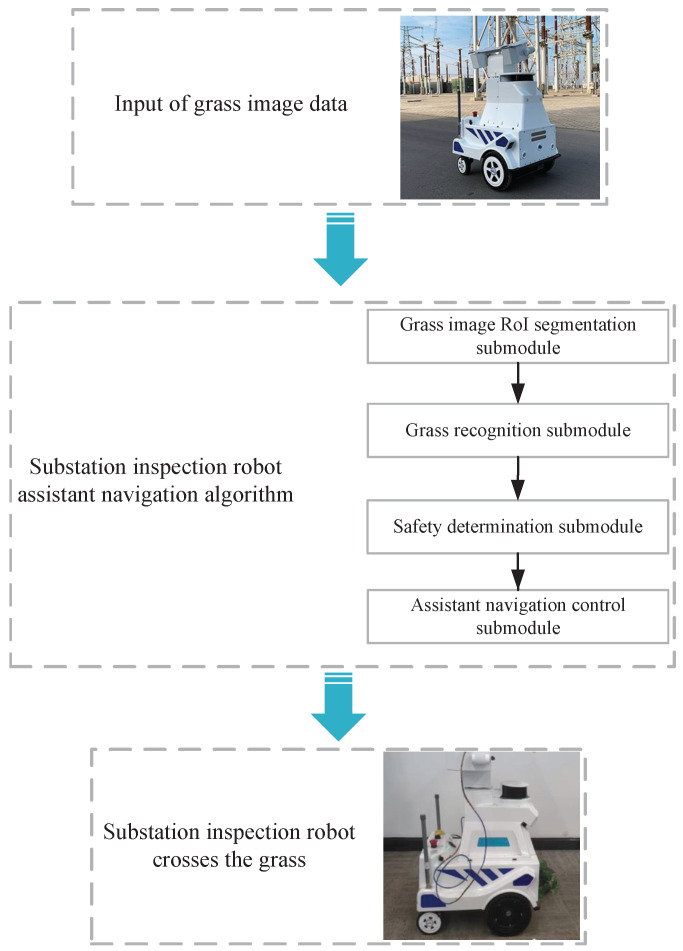
The overall scheme of smoothly cross grass areas by substation inspection robot.

In this section, the methodology proposed in this paper will be presented mainly in terms of the design of the assistant navigation algorithm. The design scheme of the inspection robot assistant navigation algorithm is mainly divided into four parts: grass image RoI (Region of Interest) segmentation submodule, grass recognition submodule, safety determination submodule, and assistant navigation control submodule. The overall scheme of grass recognition for substation inspection robot is shown in Figure 1.

**Figure 2:**
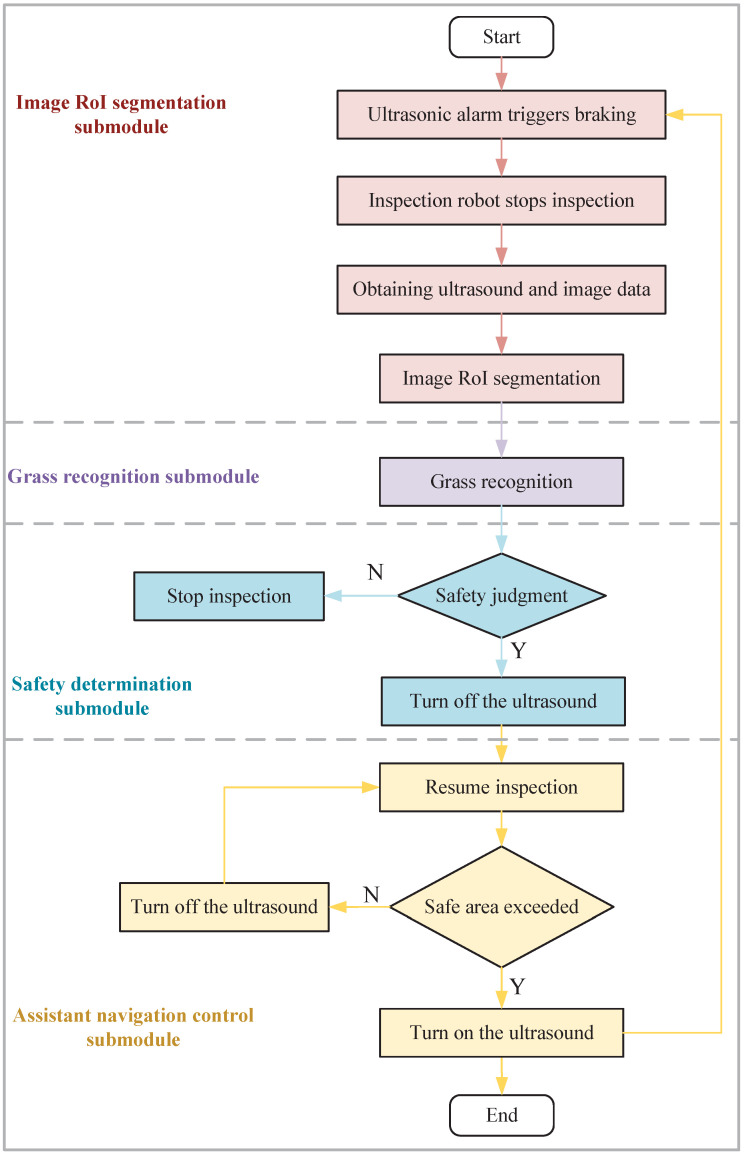
Algorithm flow of the assistant navigation.

The details of the algorithm flow are shown in Figure 2 and described as follows:

(1) Ultrasonic radar will trigger the inspection robot to brake when it detects an obstacle, and then, the robot will stop inspecting. The robot will stay in place and wait for the image RoI segmentation submodule of the auxiliary navigation algorithm to start working.
(2) Collect ultrasonic radar data and image data and perform RoI segmentation on images based on ultrasonic radar data.
(3) Recognition of the segmented image is performed using the improved model to generate detection boxes and grass category information.
(4) A safety decision method is used to determine the safety of the front of the route.
(5) Receiving the determination that the area in front of the inspection robot is safe, ultrasonic radar is blocked, and the inspection robot continues to perform the inspection task. At the same time, the assistant navigation algorithm detects the mileage data, and if the moving distance exceeds the distance between the inspection robot and the grass obstacle, the shielding ultrasonic radar is canceled, and the assistant navigation algorithm is completed.

### 2.1. Grass Image RoI Segmentation Submodule

The ultrasonic radar of the inspection robot will detect whether there are obstacles within 1 m in front. If there are obstacles, it is necessary to segment the grass image. Thus, there is a need for further processing of the grass images collected by the substation inspection robot.

The real-grass image data used in this paper is collected by a substation inspection robot. In the actual image collection process, the actual shooting distance of the camera can be up to several tens of meters away, making the grass features in the image at a longer distance unclear. As a result, the performance of the trained neural networks in grass recognition is low, so there is a need for image preprocessing. The data preprocessing method is used to blacken the unclear grass features in the image that are far away from the inspection robot.

Moreover, for deep neural networks, the small amount of data will lead to overfitting phenomena during the network training process. Therefore, to enhance the generalization of the network, data augmentation of the collected grass images is necessary. This paper uses rotation, panning, blurring, and brightness adjustment for data augmentation. The detailed results of data preprocessing and data augmentation are shown in the Section 3.

### 2.2. Grass Recognition Submodule

After RoI segmentation of the grass image, the grass of the image is recognized by the improved network, which generates the coordinate information of the detection boxes and the grass category information.

#### 2.2.1. Development of RCNN

In 2014, to address the low accuracy of the traditional sliding window method of generating detection boxes for object location within an image, the RCNN network was proposed by Ross Girshick et al. [33]. A selective search method was used in the RCNN network to generate more reliable detection boxes. This can ensure a high recall rate with fewer detection boxes selected, reduce redundant information, and make the detection boxes more accurate. Although the RCNN network has made breakthroughs in the field of object detection, there are still some shortcomings. When using CNN (Convolutional Neural Network) for feature extraction and classification, the amount of calculation and the memory space required for caching are large. Therefore, in 2015, the SPP network (Spatial Pyramid Pooling Network) was proposed by Kaiming He et al. [34]. Since this method only needs to perform one convolution operation to obtain the feature map corresponding to each detection box, it does not need to perform repeated convolution operations, which greatly enhances the detection efficiency.

Even though RCNN and SPP networks solve the problem of detection box generation and detection efficiency, they bring difficulties in network training with cumbersome steps. To solve the difficulties caused by network training, in 2015, Ross Girshack et al. combined the characteristics of RCNN and SPP networks and then proposed the Fast-RCNN network [35]. The Fast-RCNN network can train the generation of the detection boxes and the classification at the same time, simplifying the network training. With the further development of object-detection technology based on the Fast-RCNN, a Faster-RCNN network was proposed by Kaiming He et al. [36]. The RPN (Region Proposal Network) full convolutional network structure was proposed for generating detection boxes and integrated into the whole neural network structure, which removed the inaccuracy of generating the detection boxes by random search and then improved the accuracy of the object detection.

**Figure 3:**
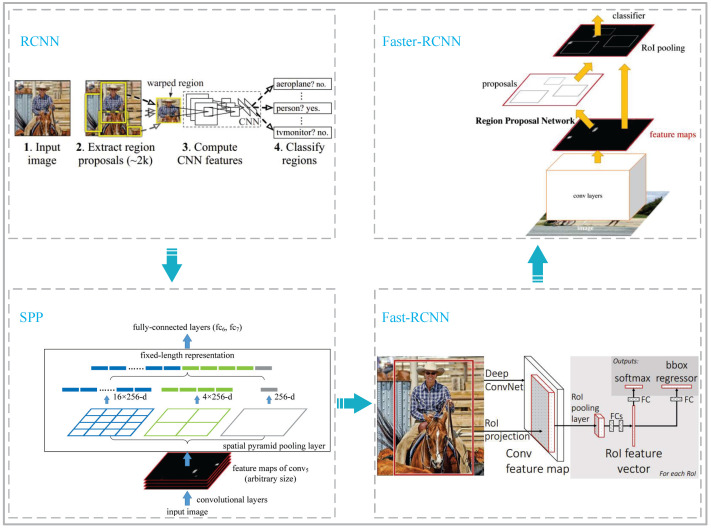
The process of developing the RCNN family.

**Figure 4:**
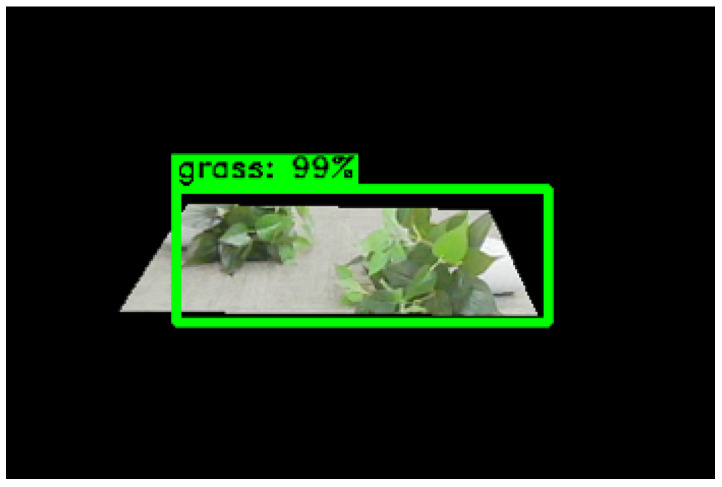
The inaccuracy of the substation grass positioning detection boxes by the Faster-RCNN network.

In summary, although the Faster-RCNN network is the most advanced in the RCNN family, the application of the Faster-RCNN object-detection network to substation grass recognition is precedent. Moreover, the experimental results of the network model with a test dataset reveal that the Faster-RCNN has a low localization accuracy for the small targets and two close targets. The process of developing the RCNN family is shown in Figure 3. The inaccuracy of the substation grass positioning detection boxes by the Faster-RCNN network is shown in Figure 4.

#### 2.2.2. Improved Faster-RCNN Network Structure

**Figure 5:**
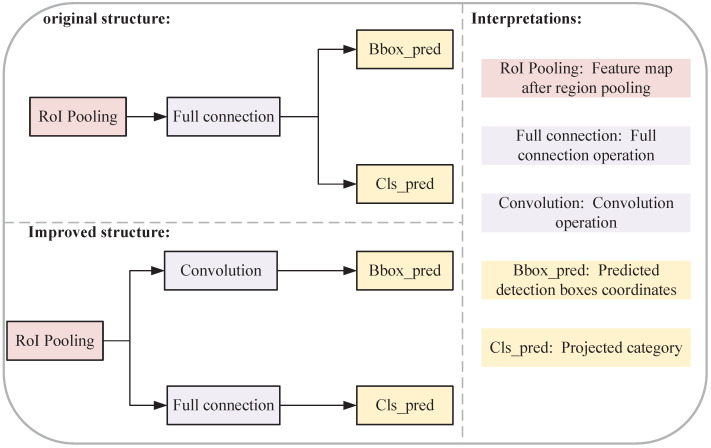
Comparison of network improvement structure.

**Figure 6:**
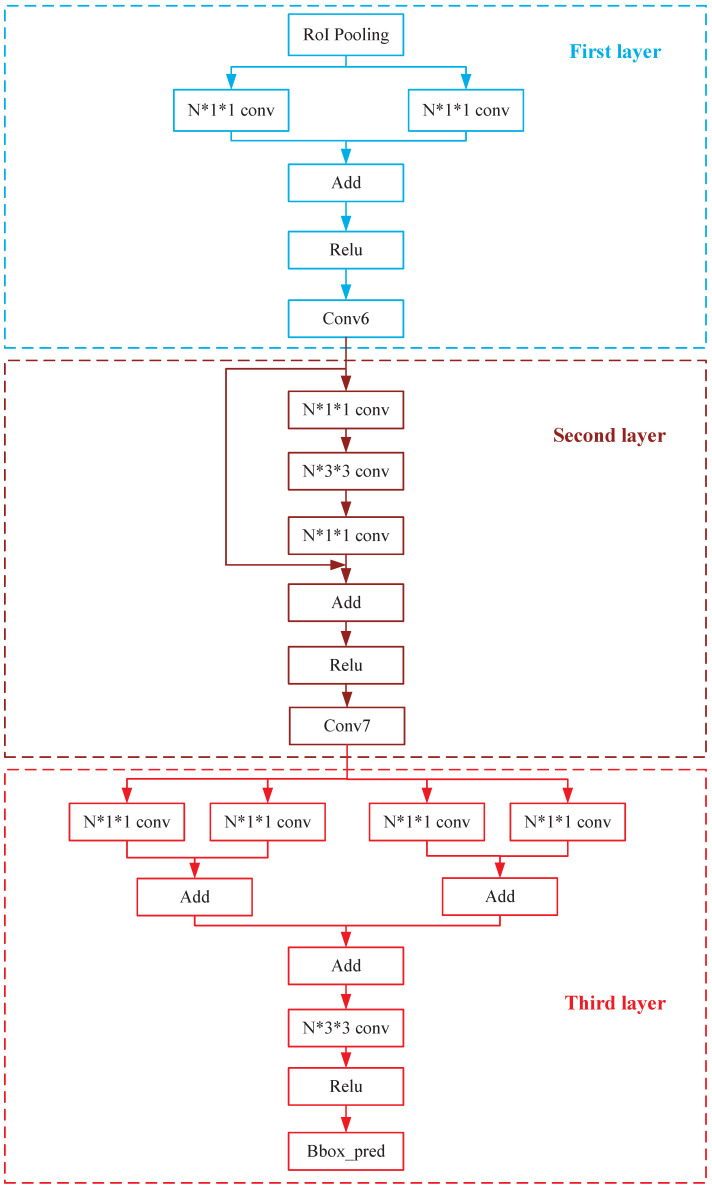
Three-layer convolutional operation structure.

To solve the problem described in Section 2.2.1, this section provides a solution based on the Faster-RCNN network. The network structure is improved as shown in Figure 5. The generated detection boxes usually belong to only one local region of the image, and the use of full connection operations makes the extracted features redundant and provides too much information. Thus, the detection boxes are generated using convolutional operations rather than full connection operations, while the object classification is still a full connection operation. Since the full connection operation in the original structured network is designed with a three-layer structure, in this paper, the added convolution operation is also designed with three different layers to ensure the accuracy of the features extracted after the convolution operation. The convolutional structure of each layer is shown in Figure 6.

From Figure 6, in the First layer, the convolution structure performs a convolution operation on the feature map RoI Pooling through a convolution kernel of size 1 * 1. Its main purpose is to increase the dimension of the feature map and extract the features of each position in the feature map. On the one hand, if the dimensionality of the convolution kernel is too high, more feature information can be extracted, and overfitting will occur. On the other hand, if the dimensionality of the convolution kernel is too low, not enough features will be available, and underfitting will occur. Therefore, it is necessary to set reasonable dimensionality parameters for the convolution kernel. In this paper, N represents the dimension parameter, and it is verified through subsequent experiments that 1024 is the optimal dimension parameter for N. To make the training optimization phase avoid gradient dispersion, the RoI Pooling feature map is operated by two convolution kernels, and then the output of two feature maps are summed to form the residual structure, fusing the two parts of the feature maps to finally output feature maps with more rich Conv6 information.

In the Second layer, the convolutional structure first performs convolutional operations on the Conv6 feature map through a convolutional kernel of size 1 * 1 to fuse the information of each dimension of the feature. Then, a convolutional kernel of size 3 * 3 is used to increase its receptive field, allowing the features to express detection boxes of varying sizes. Finally, the information from each channel is fused by a convolutional kernel of size 1 * 1. At the same time, considering the problem of gradient dispersion in the training phase, the unmanipulated Conv6 is subjected to a summing operation with the extracted feature maps to form the residual structure.

In the Third layer, the convolutional structure performs convolutional operations on the Conv7 output from the Second layer by four size 1 * 1 convolutional kernels and sums the extracted feature maps two by two, with multiple convolutional kernels to extract the features to increase the utilization of the feature maps. The last convolution operation is performed on the feature map by a convolution kernel of size 3 * 3 to enlarge the sensory field and extract richer features, which is beneficial for generating the local detection boxes task. Meanwhile, the residual structure is constructed before performing the 3 * 3 convolution, which is beneficial to the training of the network.

### 2.3. Safety Determination and Assistant Navigation Control Submodules

The safety determination method is used to analyze whether ultrasonic radar alarms are caused by the grass. There are two types of ultrasonic radar alarms: in one category, when a unilateral ultrasonic radar alarm occurs, if detection boxes appear on one side of the image and the corresponding category is grass, the ultrasonic radar alarm is considered to have been caused by the grass, and thus it is determined that the front of the inspection robot is safe; in the other category, when there is an ultrasonic radar alarm on both sides, detection boxes must appear on both sides of the image and the corresponding category is grass, and then the front of the inspection robot is determined to be safe.

Following the receipt of the determination of the safety of the area in front of the inspection robot, the alarm signal of the ultrasonic radar is blocked so that the inspection robot can perform the original inspection task. In the event of a determination that the area in front of the inspection robot is unsafe, no operation is performed without stopping working.

## 3. Experimental Results and Analysis

In this section, the experimental environment, data preprocessing, data augmentation, evaluation metrics, validation of the improved network, and implementation of the assistant navigation algorithm are described.

### 3.1. Experiments Environment and Dataset

**Figure 7:**
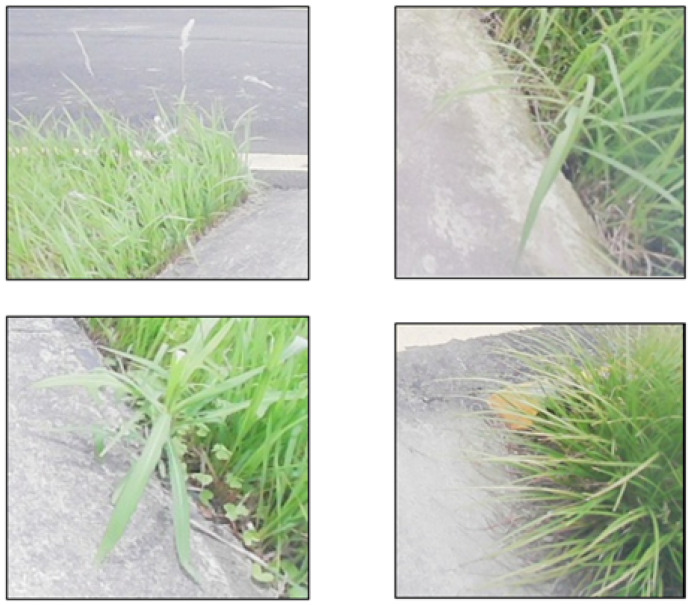
The common grass forms on the inspection robot’s route of travel.

The experimental data in this paper is derived from a real substation in China. The common grass on the inspection robot’s route of travel is shown in Figure 7. The image is collected by visible light cameras fitted to the inspection robots at different illumination levels (morning, midday, and afternoon) and in different scenarios. There is a total of 528 grass images collected in this paper to form the grass dataset, where 469 grass images are available in the training set, and 59 images are available in the test set.

In this section, all experiments are performed on the Linux operating system, Tensorflow deep-learning framework, ROS robot operating system, and substation inspection robot. The processor and RAM of the server are 3.10 GHz i5-12500H (CPU) and 32.00 GB (RAM), respectively.

**Figure 8:**
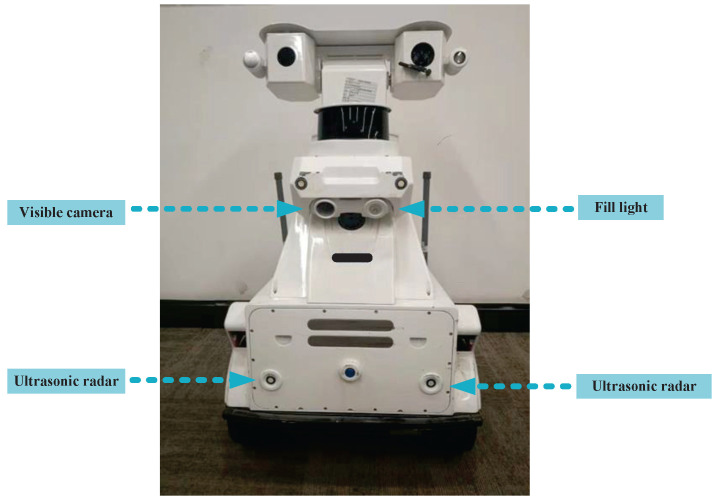
Substation inspection robot of a Chinese company.

The substation inspection robot of a Chinese company is shown in Figure 8. Substation inspection robots need to be installed with a visible camera and two ultrasonic radars. The visible camera sensor provides image information of the environment in front of the substation inspection robot for the next step of neural network detection. The ultrasonic radar measures the distance information between the grass and the substation inspection robot to control the inspection robot’s moving distance.

### 3.2. Experimental Data Preprocessing and Augmentation

**Figure 9:**
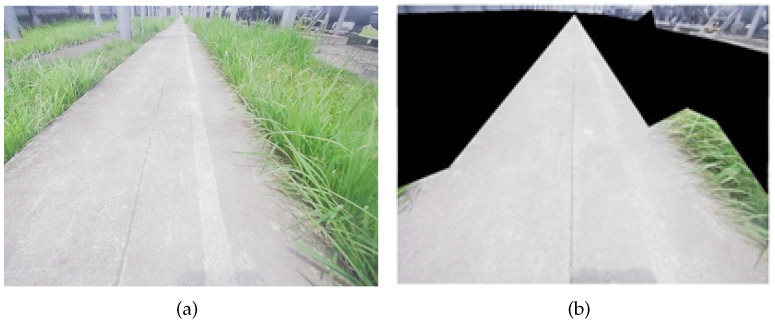
The grass image is blackened in preprocessing. (**a**) Original image; (**b**) Blackened image.

The images collected by the camera on the inspection robot are affected by light and distance, resulting in unclear grass features in some of the images. Therefore, there is a need to pre-process the collected unclear grass images. First, the unclear parts of grass features in the image are blackened to avoid sending unclear grass features into the model for training. The grass image blackening preprocessing is shown in Figure 9. Then, through the software LabelImg, parts that contain grass are labeled in each image, and the category is assigned to them as grass.

**Figure 10:**
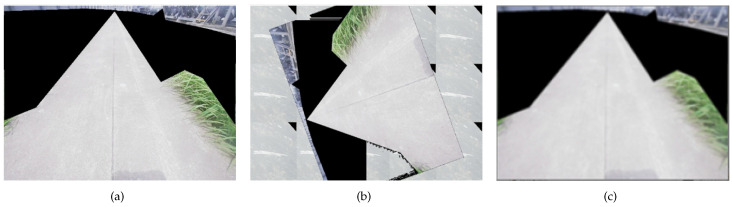
The different ways of data augmentation. (**a**) Original image; (**b**) Miscut and rotary processing; (**c**) Fuzzy processing.

Due to the fact that only 528 grass images are collected in this paper, for deep neural networks, the insufficient amount of data will lead to overfitting of the neural network and poor generalization of the model. To improve the generalization ability of the model and reduce the probability of overfitting, the grass image data are expanded by data augmentation, allowing the model to learn more grass features. The data augmentation methods are mainly used in the form of image rotation, image panning, image blurring, and image brightness adjustment, respectively. Finally, the total number of types of grass in the dataset after data augmentation is 3600, where 3240 images are in the training set, and 360 images are in the test set. The different ways of data augmentation are shown in Figure 10. The comparison of the grass dataset before and after data augmentation is shown in Table 1.

### 3.3. Evaluation Criteria

In this paper, the evaluation metrics for network accuracy are mAP (mean Average Precision) and IoU (Intersection over Union), which are described as follows.

(1) mAP
  The mAP calculation is shown in Equation (1).
  (1) $$mAP=\int_{0}^{1}P\left(R\right)dR$$
  where P is accuracy, R is recall.

(2) IoU
  The calculation of IoU is shown in Equation (2).
  (2) $$IoU=\frac{Overlap}{Union}$$
  where Overlap denotes the area of the intersection of the two detection boxes; Union denotes the total area covered by the two detection boxes.

(3) FPS
  The calculation of FPS is shown in Equation (3).
  (3) $$FPS=\frac{(Total)frames}{(Total)time}$$
  where FPS denotes the image frame rate that the network can infer per second.

### 3.4. The Performance Validation of the Improved Network

To select the basic network, Faster-RCNN [37], YOLO [38], and SSD [30] networks are tested in this paper. The results show that the SSD and YOLO networks had higher FPS but lower mAP compared to the Faster-RCNN network. The Faster-RCNN network achieved a mAP of 81.32%. Finally, Faster-RCNN was selected as the basic network of this paper. The mAP and FPS test results for different basic networks are shown in Table 2.

The substation grass data set generated above was used to train and test the network before and after the improvement, and a comparative analysis of the network before and after the improvement was conducted based on the test results. The basic network was Faster-RCNN (referred to Faster-R101), whose feature extraction network was ResNet101. In Section 2, the convolution kernel dimensions of the proposed method were set to 256, 512, 1024, and 2048, respectively. Train and test on the improved network with four different convolutional kernel dimensions. In this case, during the training process, the initial learning rate was 0.0001, the number of training iterations was 50, and 2 images per batch were trained. Finally, through the testing of the network with four different dimensional convolutional kernels, the comparisons of test results on the mAP are shown in Table 3.

As shown in Table 3, Faster-R101-Conv256, Faster-R101-Conv512, Faster-R101-Conv1024, and Faster-R101-Conv2048 are four comparison networks with different convolutional kernel dimensions. From the mAP test results of Faster-R101 and Faster-R101-Conv256, it can be seen that the mAP of the improved network structure decreases by 2.17% compared to the basic network. However, due to the low dimension of the convolution kernel of the Faster-R101-Conv256 network, which leads to insufficient expression of the features, the network suffers from underfitting, resulting in a decrease in the mAP. Therefore, the dimension of the convolutional kernel was further increased, and as can be seen from the test results of the Faster-R101-Conv512 network, the mAP was improved by 2.19% compared to the basic network, which verifies the effectiveness of using convolutional operations instead of full connection operations to optimize the performance of model detection. At this point, the convolution kernel dimension increased to 512, which improved the model’s mAP, but it was still uncertain whether the dimension was optimal.

To further determine the optimal convolution kernel dimensions, the convolution kernel dimensions increased to 1024 and 2048, respectively. From the test results of Faster-R101-Conv1024 and Faster-R101-Conv2048 networks, the mAP of Faster-R101-Conv1024 was further improved by 1.94% compared to the Faster-R101-Conv512 network. It was verified that increasing the convolutional kernel dimension to 1024 further improves the detection performance of the model and enables more features to be extracted. However, when the convolutional kernel dimension was increased to 2048, the Faster-R101-Conv2048 network showed a decrease in mAP compared to the Faster-R101-Conv1024 network. Meanwhile, the tested mAP decreased by 4.15% compared to the basic network. Thus, the Faster-R101-Conv2048 network results showed that the convolutional kernel dimension was too high for the expression of the training data features, resulting in overfitting. Based on the above analysis, the optimal dimension of the convolution kernel should be set to 1024.

**Figure 11:**
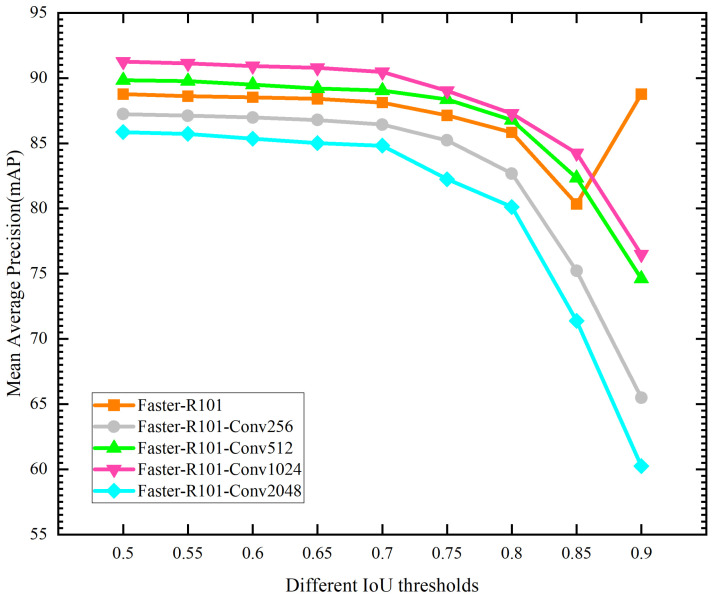
The results of different network mAP tests under IoU thresholds.

To verify the location ability of the improved network for image objects, it was necessary to calculate the mIoU of the network further. The mIoU of its location was counted by setting different thresholds. For example, if the threshold was set to 0.5 when the IoU value of the predicted detection box and its corresponding real detection box label was greater than 0.5, the detection box was considered to be accurate. The test results for positioning accuracy of different networks are shown in Table 4 and Figure 11.

In Table 4 and Figure 11, four improved networks with different convolutional kernel dimensions were compared with the basic network in terms of positioning accuracy. The experimental results show that the convolutional kernel dimension was set to 1024, and the Faster-R101-Conv1024 network had the best mAP for the network test compared to the remaining networks in the range of IoU thresholds from 0.5 to 0.9, with a mAP of 91.25% at an IoU threshold of 0.5.

**Figure 12:**
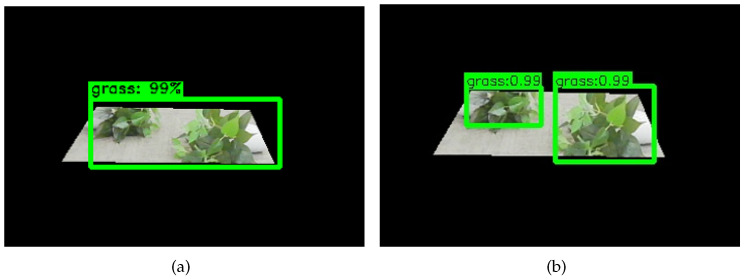
Comparison of the Faster-R101-Conv1024 network and the basic network’s actual test. (**a**) Faster-R101; (**b**) Faster-R101-Conv1024.

In summary, the convolution operation instead of the full connection operation can improve the positioning accuracy of the network and further enhance the mAP of the network. Meanwhile, the effectiveness of the convolution structure designed in this paper was verified, and the experimental results revealed that the optimal convolution kernel dimension was 1024. Thus, the improved optimal network was Faster-R101-Conv1024. The actual test results of the Faster-R101-Conv1024 network in a substation are shown in Figure 12. The actual test results show that the improved Faster-R101-Conv1024 network in this paper was more accurate in positioning the object-detection boxes.

### 3.5. The Implementation of Assistant Navigation Algorithm for Inspection Robots

This section describes the implementation of an assistant navigation algorithm for substation inspection robots. The assistant navigation algorithm mainly includes recognizing the grass in front of the substation inspection robot and assisting the substation inspection robot in crossing grass areas safely.

**Figure 13:**
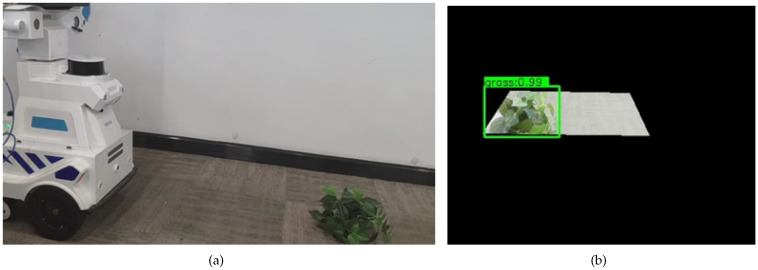

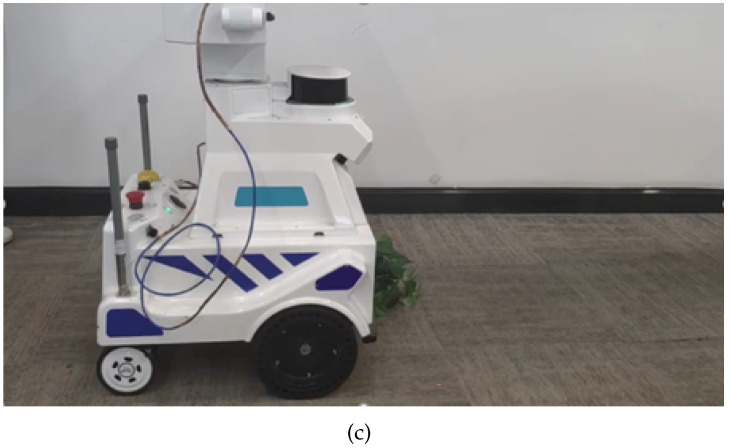
The test results of the assistant navigation algorithm in recognizing grass on the left side. (**a**) Grass is on the left; (**b**) Grass recognition result; (**c**) Safely cross the grass.

**Figure 14:**
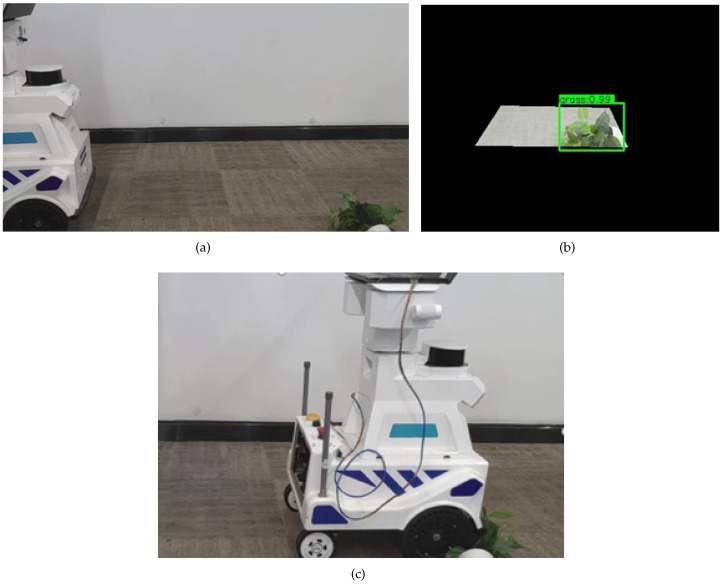
The test results of the assistant navigation algorithm in recognizing grass on the right side. (**a**) Grass is on the right; (**b**) Grass recognition result; (**c**) stately cross the grass.

**Figure 15:**
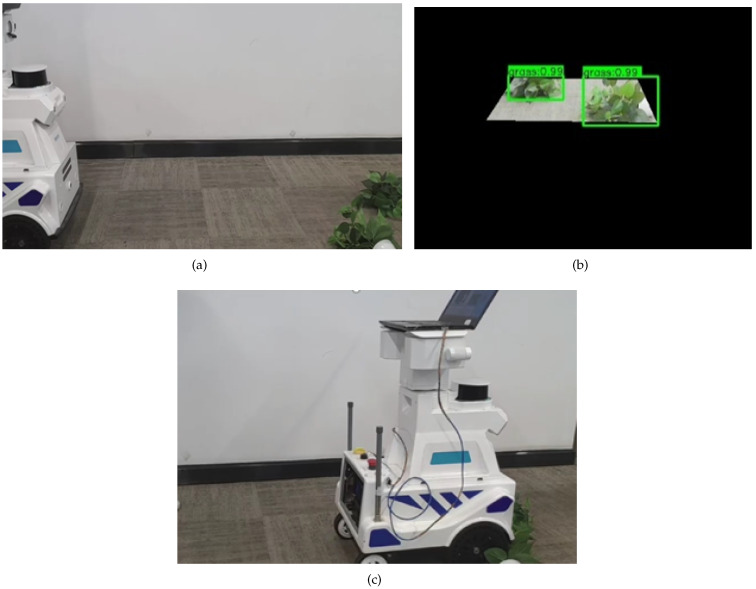
The test results of the assistant navigation algorithm in recognizing grass on both sides. (**a**) Grass is on both sides; (**b**) Grass recognition result; (**c**) safely cross the grass.

To effectively verify the assistant navigation algorithm of the substation inspection robot, according to the real situation faced by the inspection robot in the actual inspection process, the substation scenario that caused the inspection robot to stop the inspection task was simulated to test and analyze. The inspection robot will face three situations in the actual inspection process to trigger the ultrasonic radar alarm and then stop the inspection task. The three cases are grass on the left side of the inspection robot, which triggers the left ultrasonic radar alarm; grass on the right side of the inspection robot, which triggers the right ultrasonic radar alarm; and grass on both sides of the inspection robot, which triggers the bilateral ultrasonic radar alarm. The test results of the assistant navigation algorithm in recognizing grass on the left side, the right side, and on both sides are shown in Figure 13, Figure 14, and Figure 15, respectively.

In the combined three-case experiments, it was found that the assistant navigation algorithm can accurately segment the detection area of the ultrasonic radar in the image, correctly recognize the grass, and assist the substation inspection robot in crossing the grass areas safely.

## 4. Conclusions and Future Work

To solve the problems of interruption of detection tasks performed by substation inspection robots and inaccurate detection boxes during grass recognition, this paper proposes a new assistance navigation method for substation inspection robots to cross grass areas safely. First, data preprocessing and data augmentation are implemented on the collected grass image data. Second, the assistant navigation algorithm is designed to fuse the ultrasonic radar signals with visible light signals, which assist the inspection robot in crossing the grass obstacles on the route of travel safely and smoothly. In addition, to solve the inaccurate positioning of the basic network in detecting multiple objects in substation grass images, this paper improves a convolutional structure with three layers replacing the full connection structure to enhance the accuracy of detecting positioning boxes. Meanwhile, the advantages of the ResNet network are integrated to form the residual structure in the convolution. Finally, compared with several networks with different convolutional kernel dimensions, it is experimentally verified that the improved Faster-R101-Conv1024 network of this paper has the best performance in detecting multiple objects for substation grass. Compared with the basic network, the improved Faster-R101-Conv1024 network proposed in this paper improves the mAP of recognizing grass by 4.13%, and the mAP is 91.25% at IoU threshold 0.5 in the range of IoU thresholds from 0.5 to 0.9. It is also verified that the method proposed in this paper can smoothly and safely cross the grass area on the left, right, and both sides, respectively.

In future work, a transformer structure will be introduced to further improve the accuracy of detecting grass in substations. At the same time, it addresses whether the changing conditions of wet and dry grass in substations affect the performance of inspection robots.

## Figures and Tables

**Table 1:** Comparison of grass datasets before and after data augmentation.

	Total	Training Set	Test Set
Original data	528	469	59
Data augmentation	3600	3240	360

**Table 2:** The mAP and FPS test results for different basic networks.

Networks	Feature Extraction Network	mAP (%)	FPS (frame/s)
SSD	ResNet101	76.7	10
YOLO	ResNet101	63.4	18
Faster-RCNN	ResNet101	81.32	1

**Table 3:** Comparison of mAP test results for different networks.

Networks	Convolution Kernel Dimension	Learning Rate	Number of Iterations	mAP (%)
Faster-R101 (basic)	/	0.0001	50	88.41
Faster-R101-Conv256	256	0.0001	50	86.24
Faster-R101-Conv512	512	0.0001	50	90.60
**Faster-R101-Conv1024**	**1024**	0.0001	50	**92.54**
Faster-R101-Conv2048	2048	0.0001	50	84.26

**Table 4:** Comparison of mAP test results for different networks under IoU thresholds.

Networks	Different IoU Thresholds								
	0.5	0.55	0.6	0.65	0.7	0.75	0.8	0.85	0.9
	mAP (%)								
Faster-R101 (basic)	88.76	88.61	88.53	88.40	88.12	87.13	85.83	80.34	88.76
Faster-R101-Conv256	87.23	87.11	86.97	86.78	86.43	85.22	82.67	75.21	65.49
Faster-R101-Conv512	89.83	89.74	89.49	89.20	89.03	88.37	86.78	82.34	74.63
**Faster-R101-Conv1024**	**91.25**	**91.13**	**90.92**	**90.78**	**90.46**	**89.01**	**87.27**	**84.25**	**76.47**
Faster-R101-Conv2048	85.86	85.72	85.34	85.02	84.81	82.24	80.12	71.38	60.25

## Data Availability

The data used to support the findings of this study are available from the corresponding author upon request.
